# (2*E*)-2-(1,3-Benzo­thia­zol-2-yl)-3-(di­methyl­amino)­prop-2-ene­nitrile

**DOI:** 10.1107/S1600536813033266

**Published:** 2013-12-14

**Authors:** Shaaban K. Mohamed, Mehmet Akkurt, Benson M. Kariuki, Ali M. Ali, Mustafa R. Albayati

**Affiliations:** a Chemistry and Environmental Division, Manchester Metropolitan University, Manchester M1 5GD, England; b Chemistry Department, Faculty of Sccience, Minia University, 61519 El-Minia, Egypt; c Department of Physics, Faculty of Sciences, Erciyes University, 38039 Kayseri, Turkey; d School of Chemistry, Cardiff University, Main Building, Park Place, Cardiff, CF10 3AT, Wales; e Department of Chemistry, Faculty of Science, Sohag University, 82524 Sohag, Egypt; f Kirkuk University, College of Science, Department of Chemistry, Kirkuk, Iraq

## Abstract

The mol­ecular conformation of title compound, C_12_H_11_N_3_S, is almost planar [maximum deviation = 0.063 (2) Å]; an intra­molecular C—H⋯N hydrogen bond is noted. In the crystal, mol­ecules inter­act with each other *via* π–π stacking inter­actions between thia­zole rings [centroid–centroid distance = 3.7475 (9) Å] and methyl-H⋯π(C_6_) inter­actions, forming columns along the *a* axis.

## Related literature   

For various biological activities (*e.g*. anti-tumour, anti-inflammatory, anti-viral, *etc*.) of benzo­thia­zole compounds, see: Selvam *et al.* (2011 ▶); Sanja & Cvetkovic (2011 ▶); Alang *et al.* (2010 ▶); Pal *et al.* (2011 ▶); Sharma *et al.* (2010 ▶); El-Shaaer *et al.* (1997 ▶); Gupta & Raat (2010 ▶); Hutchinson *et al.* (2002 ▶); Gong *et al.* (2004 ▶); Hutchinson *et al.* (2003 ▶); Geronikaki & Theophilidis (1992 ▶); Vicini *et al.* (1990 ▶); Das *et al.* (2003 ▶); Klose *et al.* (1983 ▶); Satsangi *et al.* (1983 ▶).
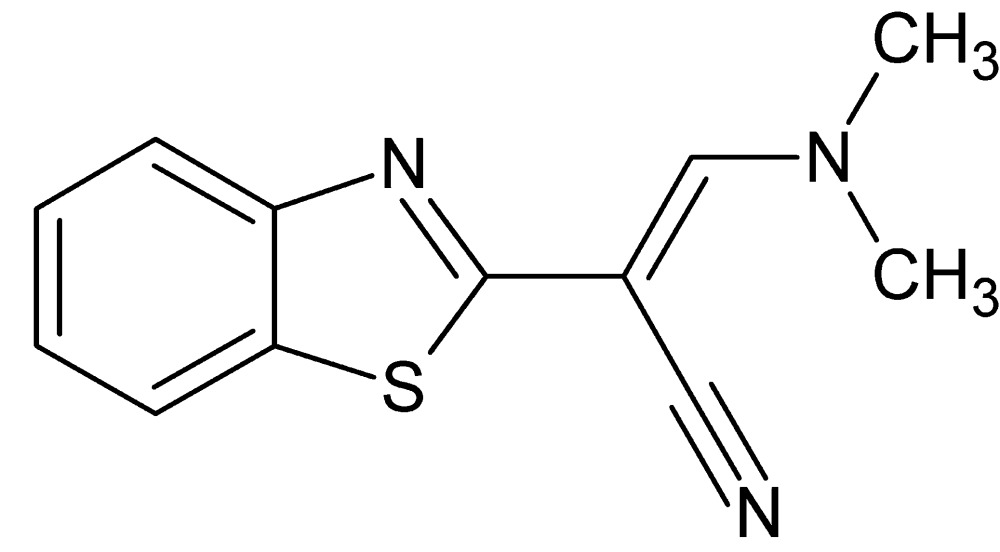


## Experimental   

### 

#### Crystal data  

C_12_H_11_N_3_S
*M* *_r_* = 229.31
Monoclinic, 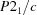

*a* = 7.3785 (2) Å
*b* = 20.1801 (4) Å
*c* = 8.2706 (2) Å
β = 112.947 (4)°
*V* = 1134.03 (6) Å^3^
*Z* = 4
Cu *K*α radiation
μ = 2.32 mm^−1^
*T* = 293 K
0.26 × 0.20 × 0.09 mm

#### Data collection  

Oxford Diffraction SuperNova (Dual, Cu at zero, Atlas) diffractometer
Absorption correction: multi-scan (*CrysAlis PRO*; Oxford Diffraction, 2013 ▶) *T*_min_ = 0.584, *T*_max_ = 0.818
4062 measured reflections
2195 independent reflections
1971 reflections with *I* > 2σ(*I*)
*R*_int_ = 0.016

#### Refinement  

*R*[*F* ^2^ > 2σ(*F* ^2^)] = 0.037
*wR*(*F* ^2^) = 0.107
*S* = 1.05
2195 reflections
147 parameters
H-atom parameters constrained
Δρ_max_ = 0.25 e Å^−3^
Δρ_min_ = −0.26 e Å^−3^

### 

Data collection: *CrysAlis PRO* (Oxford Diffraction, 2013 ▶); cell refinement: *CrysAlis PRO*; data reduction: *CrysAlis PRO*; program(s) used to solve structure: *SHELXS97* (Sheldrick, 2008 ▶); program(s) used to refine structure: *SHELXL97* (Sheldrick, 2008 ▶); molecular graphics: *ORTEP-3 for Windows* (Farrugia, 2012 ▶); software used to prepare material for publication: *WinGX* (Farrugia, 2012 ▶) and *PLATON* (Spek, 2009 ▶).

## Supplementary Material

Crystal structure: contains datablock(s) global, I. DOI: 10.1107/S1600536813033266/tk5279sup1.cif

Structure factors: contains datablock(s) I. DOI: 10.1107/S1600536813033266/tk5279Isup2.hkl

Supporting information file. DOI: 10.1107/S1600536813033266/tk5279Isup3.cml

Additional supporting information:  crystallographic information; 3D view; checkCIF report

## Figures and Tables

**Table 1:** Hydrogen-bond geometry (Å, °) *Cg*1 is the centroid of the C1–C6 ring.

*D*—H⋯*A*	*D*—H	H⋯*A*	*D*⋯*A*	*D*—H⋯*A*
C9—H9⋯N1	0.93	2.44	2.851 (2)	106
C10—H10*A*⋯*Cg*1^i^	0.96	2.77	3.549 (2)	138

Symmetry code: (i) 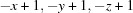.

**Fig. 1.**
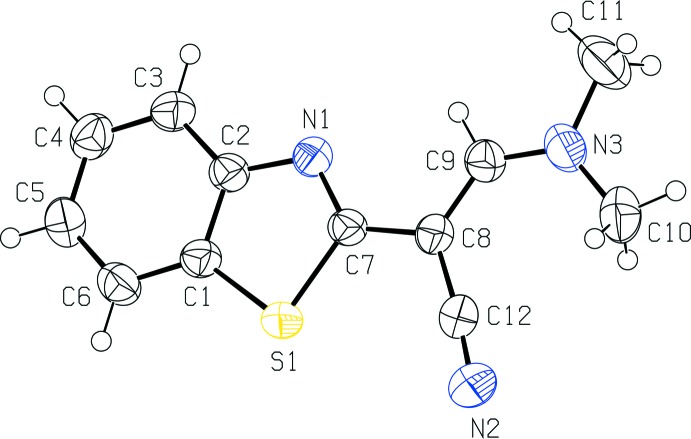
The structure of the title compound with the atom numbering scheme. Displacement ellipsoids for non-H atoms are drawn at the 50% probability level.

**Fig. 2.**
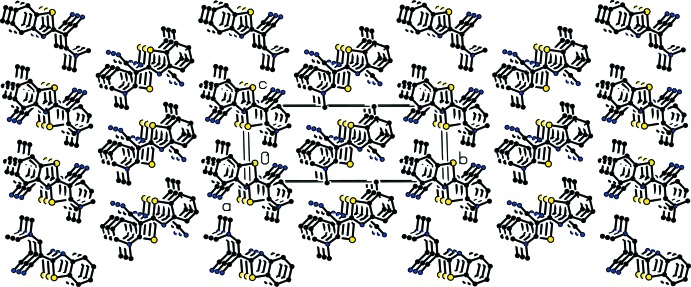
The molecular packing of the title compound viewing along the a axis. All H atoms are omitted for clarity.

## supplementary crystallographic information

### 1. Comment

It is well known that the thiazolyl group is of great importance in biological systems. In recent years, there has been considerable interest in the synthesis of substituted benzothiazolyl compounds due to their pharmacological properties such as anti-fungal (Selvam *et al.*, 2011; Sanja & Cvetkovic, 2011), anti-viral (Alang *et al.*, 2010; Pal *et al.*, 2011), anti-bacterial (Sharma *et al.*, 2010; El-Shaaer *et al.*, 1997), analgesic (Gupta & Raat, 2010), anti-tumour (Hutchinson *et al.*, 2002) and anti-tuberculosis (Gong *et al.*, 2004; Hutchinson *et al.*, 2003) activities. Moreover, such compounds have been also found to have a potent local anaesthetic activity (Geronikaki & Theophilidis, 1992; Vicini *et al.*, 1990). Anti-inflammatory, analgesic, and anti-pyretic activities for some thiazolyl and benzothiazolyl derivatives are also known (Das *et al*., 2003; Klose *et al.*, 1983; Satsangi *et al.*, 1983). Based on the above and following to our ongoing studies in synthesis of bio-active heterocyclic compounds the title compound has been prepared as a precursor for further study.

As shown in Fig. 1, title compound (I) has an almost planar conformation for non-hydrogen atoms with a maximum deviation of -0.063 (2) Å for C6.

The crystal structure is stabilized by π-π stacking interactions [*Cg*1···*Cg*1(1 - *x*, 1 - *y*, 1 - *z*) = 3.7475 (9) Å; where *Cg*1 is a centroid of the five-membered (S1/N1/C1/C2/C7) thiazole ring of the 1,3-benzothiazole ring system] between the centroids of thiazole rings of the adjacent molecules. The crystal structure has no classical hydrogen bonds. Figs. 2 show the molecular packing of (I) viewed along the a direction.

### 2. Experimental

A mixture of 1,3-benzothiazol-2-ylacetnitrile (174 mg, 1 mmol) and dimethylformamide-dimethylacetal (119 mg, 1 mmol) were taken in acetic acid (10 ml). The reaction mixture was refluxed and monitored by TLC until completion after 8 h. The reaction mixture was allowed to cool at ambient temperature and poured into ice water. The solid product was collected by filtration and recrystallized from ethanol to afford the title compound in 88% yield. Single crystals suitable for X-ray analysis were grown up on slow evaporation of ethanolic solution of the title compound at room temperature over three days. *M*.pt: 441–443 K.

### 3. Refinement

All H-atoms were refined using a riding model with C—H = 0.93 Å and *U*_iso_=1.2_eq_ (C) for aromatic-H atoms, and C—H = 0.96 Å and *U*_iso_ = 1.5*U*_eq_ (C) for methyl-H atoms.

### Crystal data

**Table:**

C_12_H_11_N_3_S	*F*(000) = 480
*M**_r_* = 229.31	*D*_x_ = 1.343 Mg m^−^^3^
Monoclinic, *P*2_1_/*c*	Cu *K*α radiation, λ = 1.54184 Å
Hall symbol: -P 2ybc	Cell parameters from 1971 reflections
*a* = 7.3785 (2) Å	θ = 4.4–72.7°
*b* = 20.1801 (4) Å	µ = 2.32 mm^−^^1^
*c* = 8.2706 (2) Å	*T* = 293 K
β = 112.947 (4)°	Block, purple
*V* = 1134.03 (6) Å^3^	0.26 × 0.20 × 0.09 mm
*Z* = 4	

### Data collection

**Table:**

Oxford Diffraction SuperNova (Dual, Cu at zero, Atlas) diffractometer	2195 independent reflections
Radiation source: SuperNova (Cu) X-ray Source	1971 reflections with *I* > 2σ(*I*)
Mirror monochromator	*R*_int_ = 0.016
ω scans	θ_max_ = 72.7°, θ_min_ = 4.4°
Absorption correction: multi-scan (*CrysAlis PRO*; Oxford Diffraction, 2013)	*h* = −7→9
*T*_min_ = 0.584, *T*_max_ = 0.818	*k* = −16→25
4062 measured reflections	*l* = −10→8

### Refinement

**Table:**

Refinement on *F*^2^	Primary atom site location: structure-invariant direct methods
Least-squares matrix: full	Secondary atom site location: difference Fourier map
*R*[*F*^2^ > 2σ(*F*^2^)] = 0.037	Hydrogen site location: inferred from neighbouring sites
*wR*(*F*^2^) = 0.107	H-atom parameters constrained
*S* = 1.05	*w* = 1/[σ^2^(*F*_o_^2^) + (0.0678*P*)^2^ + 0.1211*P*] where *P* = (*F*_o_^2^ + 2*F*_c_^2^)/3
2195 reflections	(Δ/σ)_max_ = 0.002
147 parameters	Δρ_max_ = 0.25 e Å^−^^3^
0 restraints	Δρ_min_ = −0.26 e Å^−^^3^

### Special details

**Table:**

Geometry. Bond distances, angles *etc*. have been calculated using the rounded fractional coordinates. All su's are estimated from the variances of the (full) variance-covariance matrix. The cell e.s.d.'s are taken into account in the estimation of distances, angles and torsion angles
Refinement. Refinement on *F*^2^ for ALL reflections except those flagged by the user for potential systematic errors. Weighted *R*-factors *wR* and all goodnesses of fit *S* are based on *F*^2^, conventional *R*-factors *R* are based on *F*, with *F* set to zero for negative *F*^2^. The observed criterion of *F*^2^ > σ(*F*^2^) is used only for calculating -*R*-factor-obs *etc*. and is not relevant to the choice of reflections for refinement. *R*-factors based on *F*^2^ are statistically about twice as large as those based on *F*, and *R*-factors based on ALL data will be even larger.

### Fractional atomic coordinates and isotropic or equivalent isotropic displacement parameters (Å^2^)

**Table:**

	*x*	*y*	*z*	*U*_iso_*/*U*_eq_	
S1	0.11365 (6)	0.50492 (2)	0.23607 (5)	0.0475 (1)	
N1	0.24456 (18)	0.46002 (6)	0.55620 (16)	0.0427 (4)	
N2	0.3152 (3)	0.66200 (8)	0.3024 (2)	0.0704 (6)	
N3	0.5370 (2)	0.62896 (8)	0.81666 (18)	0.0544 (4)	
C1	0.0527 (2)	0.42447 (7)	0.26748 (18)	0.0417 (4)	
C2	0.1371 (2)	0.40931 (7)	0.44773 (19)	0.0403 (4)	
C3	0.1078 (2)	0.34624 (8)	0.5022 (2)	0.0491 (5)	
C4	−0.0040 (3)	0.30080 (8)	0.3794 (2)	0.0518 (5)	
C5	−0.0886 (2)	0.31692 (9)	0.2020 (2)	0.0516 (5)	
C6	−0.0608 (2)	0.37875 (9)	0.1440 (2)	0.0501 (5)	
C7	0.2450 (2)	0.51233 (7)	0.46445 (18)	0.0388 (4)	
C8	0.3437 (2)	0.57454 (7)	0.53589 (19)	0.0411 (4)	
C9	0.4385 (2)	0.57944 (8)	0.7160 (2)	0.0459 (5)	
C10	0.5596 (3)	0.69358 (10)	0.7510 (3)	0.0643 (6)	
C11	0.6221 (4)	0.62098 (14)	1.0073 (3)	0.0817 (8)	
C12	0.3318 (2)	0.62455 (8)	0.4120 (2)	0.0491 (5)	
H3	0.16340	0.33490	0.62050	0.0590*	
H4	−0.02320	0.25870	0.41570	0.0620*	
H5	−0.16480	0.28570	0.12150	0.0620*	
H6	−0.11670	0.38950	0.02530	0.0600*	
H9	0.43210	0.54140	0.77700	0.0550*	
H10A	0.62640	0.68910	0.67250	0.0960*	
H10B	0.63510	0.72160	0.84750	0.0960*	
H10C	0.43220	0.71290	0.68920	0.0960*	
H11A	0.60670	0.57590	1.03660	0.1230*	
H11B	0.55600	0.64990	1.05890	0.1230*	
H11C	0.75950	0.63200	1.05180	0.1230*	

### Atomic displacement parameters (Å^2^)

**Table:**

	*U*^11^	*U*^22^	*U*^33^	*U*^12^	*U*^13^	*U*^23^
S1	0.0628 (3)	0.0411 (2)	0.0349 (2)	−0.0012 (2)	0.0150 (2)	0.0049 (1)
N1	0.0501 (7)	0.0382 (6)	0.0408 (6)	0.0023 (5)	0.0188 (5)	0.0008 (5)
N2	0.0975 (13)	0.0468 (8)	0.0584 (9)	−0.0053 (8)	0.0213 (8)	0.0088 (7)
N3	0.0544 (7)	0.0575 (8)	0.0464 (7)	−0.0001 (6)	0.0144 (6)	−0.0116 (6)
C1	0.0489 (7)	0.0408 (7)	0.0378 (7)	0.0023 (6)	0.0194 (6)	0.0037 (6)
C2	0.0457 (7)	0.0413 (7)	0.0365 (7)	0.0036 (6)	0.0189 (6)	0.0014 (5)
C3	0.0618 (9)	0.0453 (8)	0.0428 (8)	0.0007 (7)	0.0231 (7)	0.0065 (6)
C4	0.0642 (9)	0.0417 (8)	0.0567 (9)	−0.0033 (7)	0.0315 (7)	0.0019 (7)
C5	0.0575 (9)	0.0502 (8)	0.0518 (9)	−0.0093 (7)	0.0263 (7)	−0.0104 (7)
C6	0.0576 (9)	0.0546 (9)	0.0377 (7)	−0.0041 (7)	0.0180 (6)	−0.0027 (6)
C7	0.0443 (7)	0.0394 (7)	0.0336 (7)	0.0066 (5)	0.0162 (6)	0.0018 (5)
C8	0.0457 (7)	0.0369 (7)	0.0412 (7)	0.0041 (6)	0.0175 (6)	−0.0008 (6)
C9	0.0486 (8)	0.0454 (8)	0.0432 (8)	0.0039 (6)	0.0173 (6)	−0.0026 (6)
C10	0.0652 (10)	0.0537 (10)	0.0699 (11)	−0.0082 (8)	0.0219 (9)	−0.0178 (9)
C11	0.0848 (15)	0.0984 (17)	0.0464 (10)	−0.0053 (13)	0.0087 (9)	−0.0160 (11)
C12	0.0570 (9)	0.0379 (7)	0.0485 (8)	0.0002 (6)	0.0165 (7)	−0.0029 (6)

### Geometric parameters (Å, º)

**Table:**

S1—C1	1.7311 (15)	C7—C8	1.456 (2)
S1—C7	1.7612 (14)	C8—C9	1.380 (2)
N1—C2	1.3878 (19)	C8—C12	1.416 (2)
N1—C7	1.3008 (19)	C3—H3	0.9300
N2—C12	1.150 (2)	C4—H4	0.9300
N3—C9	1.322 (2)	C5—H5	0.9300
N3—C10	1.447 (3)	C6—H6	0.9300
N3—C11	1.461 (3)	C9—H9	0.9300
C1—C2	1.407 (2)	C10—H10A	0.9600
C1—C6	1.389 (2)	C10—H10B	0.9600
C2—C3	1.395 (2)	C10—H10C	0.9600
C3—C4	1.378 (2)	C11—H11A	0.9600
C4—C5	1.391 (2)	C11—H11B	0.9600
C5—C6	1.381 (3)	C11—H11C	0.9600
			
C1—S1—C7	89.15 (7)	C2—C3—H3	120.00
C2—N1—C7	110.61 (12)	C4—C3—H3	120.00
C9—N3—C10	124.08 (15)	C3—C4—H4	120.00
C9—N3—C11	119.72 (17)	C5—C4—H4	119.00
C10—N3—C11	116.13 (18)	C4—C5—H5	120.00
S1—C1—C2	109.23 (10)	C6—C5—H5	120.00
S1—C1—C6	129.06 (12)	C1—C6—H6	121.00
C2—C1—C6	121.71 (14)	C5—C6—H6	121.00
N1—C2—C1	115.46 (13)	N3—C9—H9	114.00
N1—C2—C3	125.84 (13)	C8—C9—H9	115.00
C1—C2—C3	118.70 (13)	N3—C10—H10A	109.00
C2—C3—C4	119.52 (14)	N3—C10—H10B	109.00
C3—C4—C5	121.02 (16)	N3—C10—H10C	109.00
C4—C5—C6	120.77 (15)	H10A—C10—H10B	109.00
C1—C6—C5	118.27 (14)	H10A—C10—H10C	109.00
S1—C7—N1	115.55 (11)	H10B—C10—H10C	110.00
S1—C7—C8	119.17 (10)	N3—C11—H11A	109.00
N1—C7—C8	125.28 (13)	N3—C11—H11B	109.00
C7—C8—C9	117.55 (13)	N3—C11—H11C	109.00
C7—C8—C12	116.15 (13)	H11A—C11—H11B	109.00
C9—C8—C12	126.30 (14)	H11A—C11—H11C	110.00
N3—C9—C8	131.03 (15)	H11B—C11—H11C	109.00
N2—C12—C8	175.21 (17)		
			
C7—S1—C1—C2	−0.33 (12)	S1—C1—C2—N1	0.48 (18)
C7—S1—C1—C6	179.26 (16)	S1—C1—C2—C3	−179.33 (12)
C1—S1—C7—C8	−179.85 (13)	N1—C2—C3—C4	179.54 (17)
C1—S1—C7—N1	0.14 (13)	C1—C2—C3—C4	−0.7 (2)
C2—N1—C7—C8	−179.91 (15)	C2—C3—C4—C5	−0.2 (3)
C7—N1—C2—C3	179.41 (16)	C3—C4—C5—C6	0.8 (3)
C2—N1—C7—S1	0.10 (19)	C4—C5—C6—C1	−0.4 (3)
C7—N1—C2—C1	−0.4 (2)	S1—C7—C8—C12	−2.3 (2)
C11—N3—C9—C8	179.2 (2)	N1—C7—C8—C9	−2.6 (2)
C10—N3—C9—C8	2.5 (3)	S1—C7—C8—C9	177.44 (12)
C6—C1—C2—C3	1.1 (2)	N1—C7—C8—C12	177.71 (15)
C6—C1—C2—N1	−179.15 (15)	C12—C8—C9—N3	0.5 (3)
S1—C1—C6—C5	179.96 (13)	C7—C8—C9—N3	−179.24 (17)
C2—C1—C6—C5	−0.5 (2)		

### Hydrogen-bond geometry (Å, º)

Cg1 is the centroid of the C1–C6 ring.

**Table:**

*D*—H···*A*	*D*—H	H···*A*	*D*···*A*	*D*—H···*A*
C9—H9···N1	0.93	2.44	2.851 (2)	106
C10—H10*A*···*Cg*1^i^	0.96	2.77	3.549 (2)	138

Symmetry code: (i) −*x*+1, −*y*+1, −*z*+1.
